# The influence of contralateral circulation on computational fluid dynamics of intracranial arteries: simulated *versus* measured flow velocities

**DOI:** 10.1186/s41747-023-00370-9

**Published:** 2023-09-22

**Authors:** SuJeong Oh, YunSun Song, HyunKyung Lim, YoungBae Ko, SungTae Park

**Affiliations:** 1 Soon Chun Hyang University College of Medicine, 59, Daesagwan-ro, Yongsan-gu, Seoul, 04401 Republic of Korea; 2 University of Ulsan College of Medicine, Asan Medical Center, 88, Olympic-ro 43-gil, Songpa-gu, Seoul, 05505 Republic of Korea; 3 Institute of Industrial Technology, 89, Yangdaegiro-gil, Ipjang-myeon, Seobuk-gu, Cheonan-si, Chungcheongnam-do 31056 Republic of Korea

**Keywords:** Carotid artery (internal), Hemodynamics, Intracranial aneurysm, Magnetic resonance angiography, Pulsatile flow

## Abstract

**Background:**

This study aimed to retrospectively evaluate the influence of contralateral anterior circulation on computational fluid dynamics (CFD) of intracranial arteries, by comparing the CFD values of flow velocities in unilateral anterior circulation with the measured values from phase-contrast magnetic resonance angiography (PC-MRA).

**Methods:**

We analyzed 21 unilateral anterior circulation models without proximal stenosis from 15 patients who performed both time-of-flight MRA (TOF-MRA) and PC-MRA. CFD was performed with the inflow boundary condition of a pulsatile flow of the internal carotid artery (ICA) obtained from PC-MRA. The outflow boundary condition was given as atmospheric pressure. Simulated flow velocities of the middle cerebral artery (MCA) and anterior cerebral artery (ACA) from CFD were compared with the measured values from PC-MRA.

**Results:**

The velocities of MCA were shown to be more accurately simulated on CFD than those of ACA (Spearman correlation coefficient 0.773 and 0.282, respectively). In four models with severe stenosis or occlusion of the contralateral ICA, the CFD values of ACA velocities were significantly lower (< 50%) than those measured with PC-MRA. ACA velocities were relatively accurately simulated in the models including similar diameters of both ACAs.

**Conclusion:**

It may be necessary to consider the flow condition of the contralateral anterior circulation in CFD of intracranial arteries, especially in the ACA.

**Relevance statement:**

Incorporating the flow conditions of the contralateral circulation is of clinical importance for an accurate prediction of a rupture risk in Acom aneurysms as the bidirectional flow and accurate velocity of both ACAs can significantly impact the CFD results.

**Key points:**

• CFD simulations using unilateral vascular models were relatively accurate for MCA.

• Contralateral ICA steno-occlusion resulted in an underestimation of CFD velocity in ACA.

• Contralateral flow may need to be considered in CFD simulations of ACA.

**Graphical Abstract:**

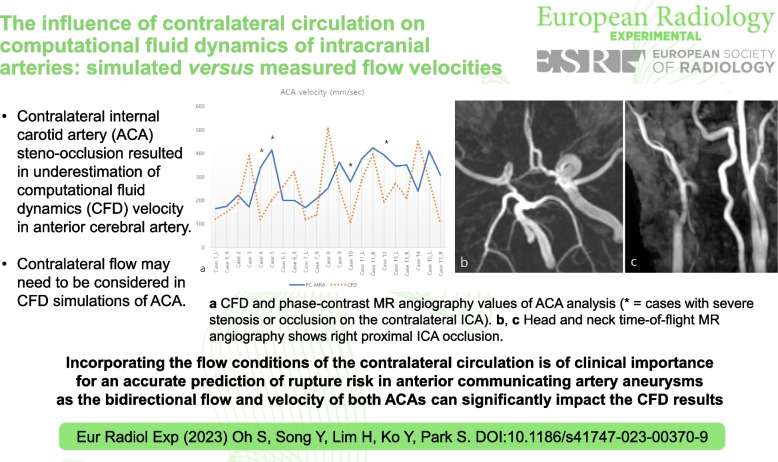

## Background

Computational fluid dynamics (CFD) of intracranial arteries is a method to predict the initiation and symptom development of vascular diseases using high-resolution three-dimensional (3D) vascular imaging. Owing to progress in the diagnostic performance of vascular imaging and increased detection of asymptomatic intracranial vascular diseases, there has been growing interest in CFD. However, its clinical application is still limited [1–3].

On the other hand, the incidence of asymptomatic cerebral aneurysms has been reported higher than previously reported [4]. Surgical clipping and vascular interventional treatments using various devices and techniques are current treatment options [5]. However, not all cerebral aneurysms need to be treated, and there are many complications following either surgical or interventional treatment [5]. For these reasons, interest in hemodynamic analysis, which can help predict the occurrence and rupture risk of cerebral aneurysms, is increasing [6].

Although the geometry of the vascular model is an important factor in CFD analysis of intracranial aneurysms [7], until now, most CFD studies have focused on the etiological analysis of the lesion with a mechanical approach, requiring costly devices and cumbersome processes. Intracranial arteries can be affected by contralateral anterior circulation and posterior circulation, due to the unique anatomical structure of the circle of Willis. However, in most of CFD studies using unilateral anterior circulation models, only ipsilateral inflow boundary condition was considered. In particular, there have been only fragmentary reports on model feasibility and inflow boundary condition in CFD of the anterior communicating artery (Acom) [8–10].

Meanwhile, software that can measure the pulsatile blood flow of complex intracranial vessels, became commercially available (Noninvasive Optimal Vessel Analysis, NOVA; VasSol, Inc., Chicago, IL, USA) [11]. NOVA uses phase-contrast MRA (PC-MRA) to measure intracranial blood flow and was reported to show good reproducibility and repeatability [11]. Based on this, we aimed to analyze the difference between the predicted intracranial blood flow from CFD and the measured value from PC-MRA and found out what anatomical conditions affect it. There are several well-known parameters in CFD of intracranial arteries including flow velocity, wall shear stress (WSS), and oscillatory shear index (OSI), we analyzed the flow velocity, which is the most important factor in CFD.

## Methods

This was an observational retrospective cross-sectional study conducted on 15 adult patients, who had undergone head TOF MRA and PC-MRA between January and December 2020 at two institutions (Asan Medical Center, Seoul, South Korea, and Sooncheonhyang University Hospital, Seoul, South Korea). Unilateral anterior circulations with severe stenosis/occlusion (*n* = 7) or coil-embolization state (*n* = 2) were excluded. Twenty-one unilateral anterior circulation models from 15 patients were included in our analysis. We received ethical approval from our institutions and informed consent was waived.

### Imaging protocol, vascular modeling, and blood flow measurement

3D surface mesh models were created using time-of-flight magnetic resonance angiography (TOF-MRA) image data obtained from two different 3-T scanners. At the Soonchunhyang University Hospital, a 750W scanner with a 24-channel head coil was used (General Electric, Boston, MA, USA) with the following technical parameters: repetition time/echo time 25/2.5 ms, flip angle 18°, acquisition voxel size 0.52 × 0.86 × 1.60 mm^3^, single-slab acquisition, acquisition time 2:30 min:s. At the Asan Medical Center, an Ingenia scanner with a 32-channel head coil was used (Philips Healthcare, Amsterdam, the Netherlands), with the following technical parameters: repetition time/echo time 18/3.5 ms, flip angle 18°, acquisition voxel size 0.6 × 0.84 × 1.2 mm^3^, single-slab acquisition, and acquisition time 4:41 min:s.

**Fig. 1:**
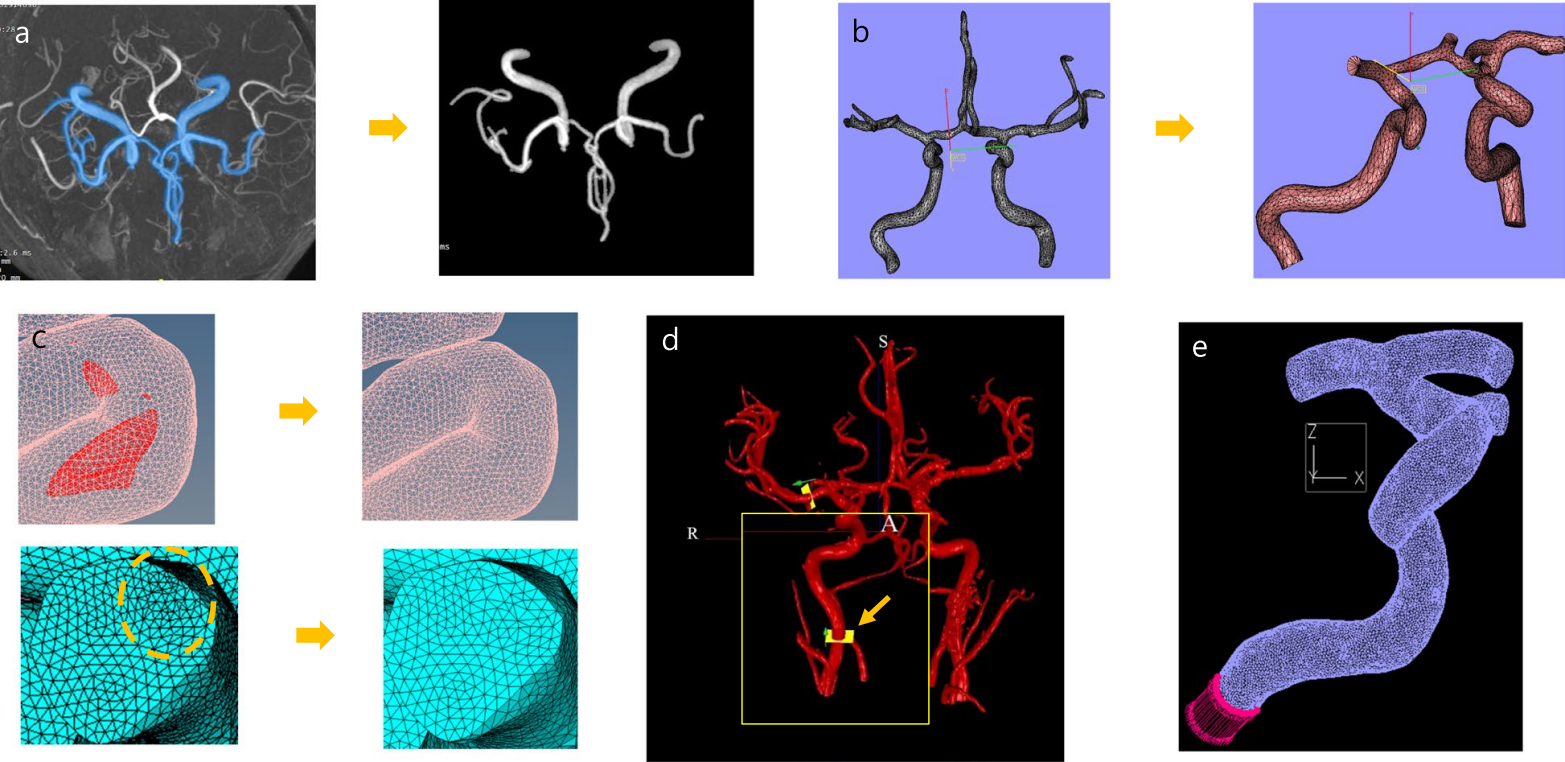
Vascular model generation and post-processing.** a** Three-dimensional surface model was extracted from TOF-MRA. **b** Small branches of triangular surface mesh were edited. **c** Inadequate triangular meshes were edited and removed. **d** Pulsatile flow data were obtained from two-dimensional PC-MRA with the aid of NOVA (arrow) using the TOF-MRA template. **e** Model-specific flow input was given parallel to the vessel centerline. *PC-MRA* Phase-contrast magnetic resonance angiography, *TOF-MRA* Time-of-flight magnetic resonance angiography

Aquarius iNtuition (Terarecon, Foster City, California, USA), a medical software program designed for medical image visualization and analysis, was used to create the 3D surface mesh models. Magics RP (Materialise, Leuven, Belgium) was used for shape editing such as removal of small (< 1 mm) blood vessels and removal of kissing artifacts from adjacent blood vessels. The surface mesh was edited and the volume mesh model was generated using Hypermesh (Altair Computers and Structures, Auckland, New Zealand) (Fig. 1).

For PC-MRA analysis, the abovementioned same software (NOVA) was utilized in both hospitals to obtain the blood flow velocities with the vendor-provided protocol (repetition time/echo time 10–15/4–7 ms, flip angle 15°, number of excitations 4, slice thickness 3 mm for intracranial arteries and 5 mm for neck arteries, field of view 140 mm for intracranial arteries and 180 mm for neck arteries, matrix 256 × 192 for intracranial arteries and 256 × 128 for neck arteries). Although different MR scanners were used in the two hospitals (Soonchunhyang University Hospital, General Electric 750W with 24-channel head coil; Asan Medical Center, Philips Ingenia with 32-channel head coil), the protocol used for PC-MRA analysis was suggested by the vendor and remained consistent in both hospitals, as described above. NOVA software utilized a 3D surface mesh model, generated from TOF MRA, for determining the perpendicular scan plane to vessels of interest. Velocity encoding was automatically adjusted by the NOVA software to ensure that aliasing did not occur for high velocities within the selected velocity range for phase encoding of individual vessels. Blood flow velocities were obtained for the anterior cerebral artery (ACA), middle cerebral artery (MCA), and internal carotid artery (ICA) from PC-MRA.

### Computational fluid dynamics analysis

CFD was performed using commercial software ADINA (ADINA R&D, Inc., Watertown, MA, USA). The blood flow was assumed to be laminar, viscous, Newtonian, and incompressible (viscosity 0.004 N-s/m^2^, density 1100 kg/m^3^). Inflow boundary condition was given as a model-specific pulsatile flow of ICA obtained from PC-MRA and the blood flow direction was given perpendicular to the inlet surface using a skew system. For postprocessing, average blood flow velocity was calculated in the ACA A1 segment and MCA M1 segment using the ADINA postprocessing module.

### Statistical analysis

Normal data distribution was checked using Shapiro-Wilk test (*p* < 0.05). Mean and standard deviation SD of the CFD and PC-MRA values of ACA and MCA velocities were calculated. Mean differences between the CFD and PC-MRA values were also calculated with standard deviation. Correlation between the CFD and PC-MRA values of ACA and MCA velocities were evaluated with Spearman correlation test and Bland-Altman analysis. All data analyses were performed with SPSS version 29 (IBM, Armonk, NY, USA); *p* values lower than 0.05 were considered to indicate a statistical significance.

## Results

Twenty-one unilateral anterior circulation models from 15 patients were analyzed. Relevant anatomic features are shown in Table 1.

**Table 1:** Relevant anatomic features

Case	Relevant anatomic features
1	None
2	Contralateral carotid bulb stenosis Contralateral ACA A1 hypoplasia
3	Coil embolization state (contralateral p-com)
4	Contralateral ICA occlusion
5	Contralateral ICA occlusion
6	None
7	None
8	Coil embolization state (contralateral MCA M2)
9	Contralateral MCA M1 moderate stenosis
10	Contralateral ICA severe stenosis
11	None
12	Contralateral ICA severe stenosis
13	None
14	Contralateral ACA A1 stenosis
15	None

*ACA* Anterior cerebral artery, *ICA* Internal carotid artery, *MCA* Middle cerebral artery, *p-com* Posterior communicating artery

In both ACA and MCA analyses, the CFD values were generally lower than the PC-MRA values. In MCA analysis, the CFD and PC-MRA values showed differences of more or less than 20%, while the differences ranged from 6 to 65% for ACA analysis (Table 2, mean difference 31.4 *versus* 59.4 mm/s).

**Table 2:** MCA and ACA velocities: measured values (NOVA) and simulated values (ADINA)

Case	CFD	PC-MRA		CFD	PC-MRA	
	MCA velocity (mm/s) (A)	MCA velocity (mm/s) (B)	B/A	ACA velocity (mm/s) (C)	ACA velocity (mm/s) (D)	D/C
1 left	174.77	209.72	1.20	165.65	120.81	0.73
1 right	232.11	274.75	1.18	175.17	152.07	0.87
2	225.94	187.78	0.83	222.53	190.23	0.85
3	320.49	263.33	0.82	172.16	392.16	2.28*
4	380.58	359.77	0.95	340.28	120.27	0.35*
5	322.68	174.00	0.54	414.88	202.52	0.49*
6 left	436.37	545.31	1.25	201.15	261.15	1.30
6 right	434.03	600.24	1.38	201.15	322.31	1.60
7 left	290.83	261.43	0.90	170.17	117.87	0.69
7 right	378.91	277.23	0.73	209.24	139.22	0.67
8	389.13	236.14	0.61	251.44	507.52	2.02*
9	378.19	262.94	0.70	362.78	252.69	0.70
10	288.03	271.26	0.94	279.06	107.48	0.39*
11 left	471.88	452.78	0.96	377.85	287.79	0.76
11 right	623.33	525.42	0.84	424.10	397.88	0.94
12	302.83	220.94	0.73	393.82	192.26	0.49*
13 left	454.91	431.62	0.95	346.40	272.31	0.79
13 right	369.97	299.09	0.81	350.58	210.11	0.60
14	278.64	262.57	0.94	239.00	134.37	0.56
15 left	589.07	481.48	0.82	411.08	282.90	0.69
15 right	406.44	492.83	1.21	307.91	104.20	0.34

CFD and PC-MRA values showed lower differences for MCA than for ACA (mean difference 32.59 *versus* 64.97 mm/s). In the ACA analysis, the CFD values were significantly lower than those obtained from PC-MRA in the case of severe stenosis or occlusion in the contralateral ICA (cases 4, 5, 10, and 12). Meanwhile, in two cases (3 and 8), the CFD values of ACA velocities were much higher than those obtained from PC-MRA; in both of them, coil embolization had been performed on the contralateral side

*ACA* Anterior cerebral artery, *CFD* Computational fluid dynamics, *ICA* Internal carotid artery, *MCA* Middle cerebral artery, *PC-MRA* Phase-contrast magnetic resonance angiography

* reflects the ones with significant difference between the measured and simulated ACA velocities

**Fig. 2:**
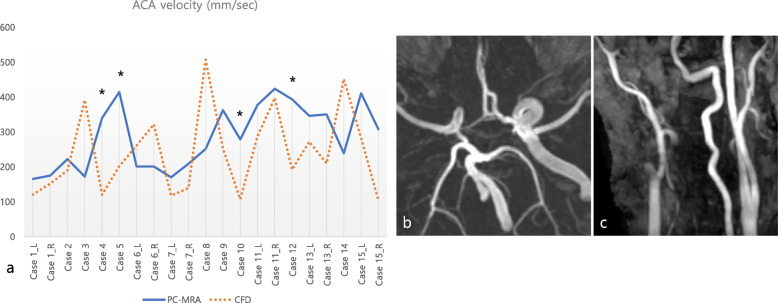
CFD and PC-MRA values of ACA analysis. **a** CFD and PC-MRA values of ACA analysis (asterisk: cases with severe stenosis or occlusion on the contralateral ICA). **b**, **c** Head and neck TOF MRA in case 5 show right proximal ICA occlusion. *ACA* Anterior cerebral artery, *CFD* Computational fluid dynamics, *ICA* Internal carotid artery, *PC-MRA* Phase-contrast magnetic resonance angiography, *TOF-MRA* Time-of-flight magnetic resonance angiography

In ACA analysis, the CFD values were significantly lower (less than 50%) than the PC-MRA values when there was severe stenosis or occlusion in the contralateral ICA (cases 4, 5, 10, 12; Figs. 1 and 2). On the other hand, when there was no significant stenosis in both ICAs and both ACAs were similar in diameter, differences between the CFD and PC-MRA values were relatively lower (from 6 to 40%). Meanwhile, in two cases, the CFD values of ACA velocities were much higher than the PC-MRA values (cases 3 and 8): in both cases, coil embolization had been performed on the contralateral side.

**Fig. 3:**
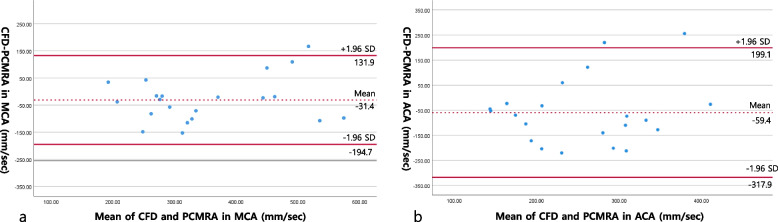
Bland-Altman plots comparing the level of agreement between PC-MRA and CFD values in MCA and ACA (mm/s). *ACA* Anterior cerebral artery, *CFD* Computational fluid dynamics, *MCA* Middle cerebral artery, *PC-MRA* Phase-contrast magnetic resonance angiography

Using Spearman correlation test, the correlation between the CFD and PC-MRA values was better for MCA analysis than for ACA analysis (Spearman coefficient 0.773 *versus* 0.282, respectively, *p* < 0.001, *p* = 0.216). Using Bland-Altman analysis, 95% limits of agreement was narrower for MCA analysis than for ACA analysis (–194.7 to 131.9 and –317.9 to 199.1, respectively; Table 3 and Fig. 3).

**Table 3:** Mean difference, 95% limits of agreement, and Spearman correlation between phase-contrast magnetic resonance angiography and computational fluid dynamics values for MCA and ACA

	Mean difference ± standard deviation	95% limits of agreement	Spearman coefficient	^*^p value
MCA	–31.4 ± 83.3	–194.7 to 131.9	0.773	< 0.001
ACA	–59.4 ± 131.9	–317.9 to 199.1	0.282	0.216

*ACA* Anterior cerebral artery, *MCA* Middle cerebral artery.

^*^p value from Spearman correlation

## Discussion

With recent advances in vascular imaging, the detection of asymptomatic cerebral aneurysms is increasing [12]. However, asymptomatic cerebral aneurysms have a relatively low rupture risk and rarely result in rupture [12, 13]. In addition, since various complications can occur following surgical or interventional treatment [14–18], it is necessary to evaluate the benefit of treatment along with the rupture risk of a cerebral aneurysm [19]. Considering this background, many studies have been conducted on CFD, which is expected to be helpful in predicting the rupture risk of a cerebral aneurysm or the outcome of an interventional procedure [1, 2, 6, 8–10, 19]. However, in CFD-related studies that evaluated various hemodynamic parameters using 3D vascular models, analysis regarding the anatomical differences of the vascular model has been done much. In particular, only a few studies have considered the effect of contralateral anatomy [7–10]. In this study, we aimed to analyze the influence of anatomical conditions of the vascular models by the means of comparing the CFD results with the actual values measured from PC-MRA.

For MCA analysis, CFD simulations were relatively accurate and showed more or less than 20% differences from PC-MRA values. It suggests that blood flow in MCA could be better simulated than in ACA on CFD using unilateral vascular models. It is presumed because MCA is less influenced by collateral supplies, compared to ACA. Thus, there would be no great difficulty in simulating aneurysms in MCA using CFD.

For ACA analysis, CFD values were significantly lower than PC-MRA values when there was severe stenosis or occlusion in contralateral ICAs (cases 4, 5, 10, and 12), probably because the unilateral vascular models do not reflect the increased flow to supply the reduced contralateral flow though Acom. Prominent Acoms were observed in those models. Case 2 had severe stenosis in the contralateral ICA but also hypoplasia of the contralateral ACA A1 segment and the simulated ACA velocity was relatively accurate, probably because there was little blood flow through the Acom, resulting in less difference between the CFD and the PC-MRA values, compared to other cases with contralateral ICA stenosis. On the other hand, in cases 9 and 14, there were stenoses in the contralateral intracranial vessels (MCA M1 segment and ACA A1 segment, respectively), and unlike cases with contralateral ICA stenosis, there was no significant difference between the CFD and the PC-MRA values.

CFD simulations of Acom aneurysms can be affected by contralateral anterior circulation through the circle of Willis and some of previous studies have taken this into consideration. Castro et al. [9] found that contralateral inflow boundary conditions affected the CFD results of Acom aneurysms although they did not use patient-specific data. Our study also suggests that CFD of the lesions at locations that are prone to be affected by contralateral anterior circulation requires bilateral inflow boundary conditions.

The bidirectional flow and accurate velocity of both ACAs can significantly impact the CFD results, particularly in terms of the flow shape and the location of low WSS, both of which play a crucial role in predicting the rupture risk of Acom aneurysms. Therefore, incorporating the flow conditions of the contralateral anterior circulation is of clinical importance to achieve a more accurate prediction of rupture risk in Acom aneurysms.

Finally, in this study, the measured ACA and MCA velocities showed a wide range of values, which is consistent with previous reports that showed a wide range of flow velocities in intracranial arteries [20–22]. Therefore, using patient-specific data is thought to be more reliable in CFD to obtain quantitative results such as WSS and OSI.

This study had several limitations. First, this study has a retrospective study design with a relatively small sample size. Second, there are intrinsic limitations in measuring intracranial arterial velocities using PC-MRA including partial volume effect, intravoxel phase dispersion, and displacement artifact [23]. However, PC-MRA is the only method with verified reproducibility in measuring intracranial blood flow velocities, so far. Third, intracranial arteries in posterior circulation were not taken into consideration despite a relatively high rupture risk and clinical importance of posterior circulation aneurysms [24, 25]. Fourth, other hemodynamic parameters known to be related to rupture risk of intracranial aneurysms, including WSS and OSI [2, 3, 6, 26]), were not directly measured and evaluated. However, these parameters can also be derived using specific equations, and the assessment of flow velocities plays an essential role in their calculation. Finally, this study was designed with the underlying assumption of laminar flow and Newtonian incompressible flow for blood flow. This assumption cannot completely describe real blood flow; however, to date, most clinical papers have been based on this assumption [27].

This study adds another point of view on many technical, logical limitations of CFD. We used patient-specific models and flow velocities, trying to compare the CFD results with the measured values. Our results might serve as evidence to increase confidence in CFD.

In conclusion, in the CFD simulation of ACA flow velocity using the unilateral anterior circulation model, the CFD values can be significantly lower than the PC-MRA values when there is severe stenosis or occlusion in the contralateral ICA. It may be necessary to consider the flow condition of the contralateral anterior circulation when performing CFD of intracranial arteries.

## Data Availability

The datasets used and/or analyzed during the current study are available from the corresponding author on reasonable request.

## Abbreviations

3D: Three dimensional
ACA: Anterior cerebral artery
Acom: Anterior communicating artery
CFD: Computational fluid dynamics
ICA: Internal carotid artery
MCA: Middle cerebral artery
NOVA: Noninvasive optimal vessel analysis
OSI: Oscillatory shear index
PC-MRA: Phase-contrast-magnetic resonance angiography
TOF-MRA: Time-of-flight magnetic resonance angiography
WSS: Wall shear stress
